# Awareness campaigns and strengthened prevention as alternatives to banning: Preventing zoonotic diseases from wildlife in the Democratic Republic of Congo

**DOI:** 10.1371/journal.pone.0327590

**Published:** 2025-07-01

**Authors:** Marc K. Yambayamba, Chloe Clifford Astbury, Hélène Carabin, Eduardo Gallo-Cajiao, Kirsten M. Lee, Désiré K. Mashinda, Justin M. Masumu, Simon R. Ruegg, Tarra L. Penney, Mala Ali Mapatano

**Affiliations:** 1 Section of Epidemiology, Vetsuisse Faculty, University of Zurich, Zürich, Switzerland; 2 Department of Epidemiology and Biostatistics, University of Kinshasa School of Public Health, Kinshasa, Democratic Republic of the Congo; 3 School of Global Health, York University, Toronto, Ontario, Canada; 4 Dahdaleh Institute for Global Health Research, York University, Toronto, Ontario, Canada; 5 Global Strategy Lab, York University, Toronto, Ontario, Canada; 6 Faculté de Médecine Vétérinaire, Université de Montréal, Saint-Hyacinthe, Québec, Canada; 7 Groupe de Recherche en Épidémiologie des Zoonoses et Santé Publique (GREZOSP), Saint-Hyacinthe, Quebec, Canada; 8 School of Public Health, Université de Montréal, Montréal, Quebec, Canada; 9 Department of Human Dimensions of Natural Resources, Warner College of Natural Resources, Colorado State University, Fort Collins, Colorado, United States of America; 10 Faculté de Médecine Vétérinaire, Université Pédagogique Nationale, Kinshasa, Democratic Republic of the Congo; 11 Institut National des Recherches Biomédicales, Kinshasa, Democratic Republic of the Congo; 12 Network for Ecohealth and One Health, Basel, Switzerland; 13 Department of Nutrition, University of Kinshasa School of Public Health, Kinshasa, Democratic Republic of the Congo; Government College of Engineering, Keonjhar, INDIA

## Abstract

**Background:**

The Democratic Republic of Congo (DRC) faces a rising frequency of emerging infectious diseases outbreaks such as Ebola and Mpox. Wild meat consumption is considered a risk factor due to increased contact with wild animals. This study aimed to identify sociodemographic characteristics associated with wild meat consumption, assess the perceived risk of infectious diseases among consumers, and investigate attitudes towards selective measures to control disease spillover from wildlife.

**Methods:**

A cross-sectional survey was conducted from June to August 2022 in four major cities: Kinshasa (Kinshasa), Kindu (Maniema), Lodja (Sankuru), and Boende (Tshuapa). Adults aged 18 years or older participated through a pre-tested questionnaire. Data included demographic characteristics, wild meat consumption behaviors, zoonotic disease risk perception, and potential human-wildlife disease prevention measures. The latter included measures such as law enforcement, education, and awareness campaigns, investing in disease prevention, strengthening response, and banning wild meat. Multivariable logistic regression was used to analyze associations between demographics, consumption, and risk perception.

**Findings:**

Of 2,163 respondents, 59% were male, and 38% were aged 26–35. Wild meat consumption was reported by 86%. The main reason for consumption across cities was the meat taste (76%). Overall, only 36% of wild meat consumers perceived themselves to be at risk of a zoonotic disease. The highest risk perception was reported to be as high as 92% in Boende. Residents of Lodja had higher odds of wild meat consumption (OR: 11.4, CI: 6.35–21.40) compared to Kinshasa followed by those living in Kindu (1.61, 1.09–2.37), this association was also statistically significant in Boende. Risk perception was higher in Boende (OR: 5.26, CI: 1.72–15.0) and lower in Lodja (OR: 0.25, CI: 0.09–0.60) compared to Kinshasa. Knowing a family member or a relative infected with zoonotic disease increased risk perception (OR: 5.55, CI: 2.29–13.40). More than 70% of respondents supported measures such as awareness campaigns, increased disease prevention budgets, and law enforcement. Banning wild meat consumption was least supported across cities.

**Conclusion:**

The findings highlight that wild meat consumption is quite homogenous with regards to sociodemographic characteristics, only the city of residence emerged as a significant factor. However, the risk perception is very low. Increased awareness campaigns and biosafety measures along the value chain would contribute to the prevention of zoonotic diseases originating from wildlife.

## Background

In recent years, the world has faced emerging infectious diseases, such as Ebola, COVID-19, Mpox and Middle East Respiratory Syndrome (MERS) which have significantly impacted livelihoods and economies [1–3]. More than 70% of these diseases have originated from direct contact between humans and wildlife either through trade and consumption or indirectly through interactions between livestock and wildlife [4,5]. Human activities, such as encroachment into wildlife habitat, land use change, increasing demand for animal protein, and globalisation, have been identified as drivers of zoonotic spillover and spread [1,6–9]. It is estimated that more than one billion contacts between human and wildlife occur annually through hunting, trapping and butchering wildlife; transport of live animals and animal products; selling of wildlife meat at markets; as well as consumption and use of wild meat products [1,10]. The One Health High Level Expert Panel of the Quadripartite (OHHLEP) emphasizes that successful spillover prevention requires a proactive response, one that addresses the drivers of disease emergence including wildlife trade and wild meat consumption [6,8].

Wildlife hunting, trade, and consumption are well-documented practices in the Congo-basin region, an irreplaceable global biodiversity hotspot [11], where socio-economic hardship is common, and law enforcement capacity is limited [12,13]. It is estimated that 86% of harvested wild meat is used for household consumption in the Congo basin with an estimated one million metric tons consumed each year [14,15], while the remaining proportion provides income for many households [16]. In sub-Saharan Africa, rapid urbanization has increased food demand in urban areas, prompting a shift in food retail markets from rural to urban regions [9,16–19]. Driven by these changes, increases in the human-animal interface and increases in infectious disease emergence, such as HIV-1, Ebola and Mpox, has been noted in the region [3,17].

The Democratic Republic of Congo (DRC) is part of the Congo basin. Conflicts affecting some of the protected areas in the country exacerbates economic hardship and hinders biodiversity conservation efforts, leading to increased wild meat trade and consumption [18–21]. This increases contacts with wildlife representing an opportunity for disease spillover. As of 2023, the country has faced fifteen Ebola outbreaks since 1976, originating mostly in rural areas where limited detection capacity causing delays in response and resulting in both direct and indirect consequences [22,23]. The 2018−19 Ebola outbreak, which affected North Kivu, South Kivu and Ituri, was long lasting and difficult to control as it occurred in a conflict region with lack of trust in the health system, posing challenges to response activities, such as attacks towards the frontline personnel [24,25]. Since early 2022, the country is facing an unpreceded Mpox outbreak with cases moving out of the traditional three endemic provinces (Tshuapa, Tshopo and Sankuru) to more than 22 provinces [26]. These outbreaks have had a high impact on a health system already facing other challenges, including other communicable and non-communicable diseases and resource constraints. Strengthening surveillance and detection were listed as key recommendations of the Joint External Evaluation of the International Health Regulation capacities (JEE-IHR) conducted in 2018 [27]. Understanding factors associated with wild meat consumption (e.g., motivations, species consumed) and associated risk perception would inform public health authorities with essential evidence to support the design of appropriate prevention strategies, including improved surveillance. Most studies conducted to understand the behaviour along the wild meat value chain have used a qualitative design to collect data from hunters and vendors and focused on risky behaviour along the value chain [17,28,29]. Furthermore, there is limited data from the general population regarding factors associated with wild meat consumption and the perceived risk of emerging infectious diseases in the country. Against this backdrop, this study aims to: (i) evaluate the magnitude, taxonomic composition, and sourcing of the consumed meat, (ii) assess the perceived risk of infectious diseases among consumers, iii) identify sociodemographic characteristics associated with wild meat consumption, and (iv) examine attitudes towards targeted measures to prevent disease spillover from wildlife.

## Materials and methods

### Study setting and study design

**Fig 1:**
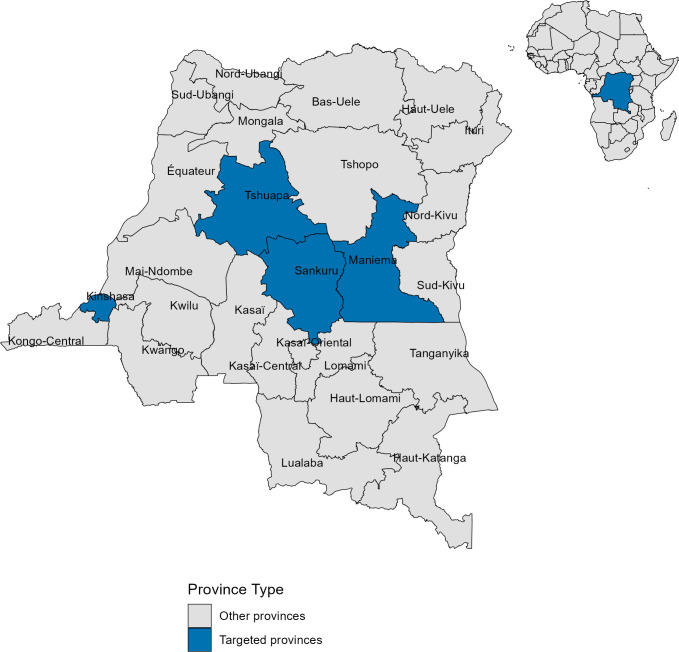
Map of the Democratic Republic of the Congo showing study provinces. Provinces highlighted in blue—Kinshasa, Tshuapa, Sankuru, Maniema, and Tshopo—were targeted for data collection and analysis (Map created using in R software using open data).

We conducted a cross-sectional study in the DRC from mid-June to mid-August 2022. With 26 provinces, the country is the second largest in Africa in terms of area, with an estimated human population close to 100 million inhabitants [30,31]. The four cities of Kinshasa (Kinshasa), Kindu (Maniema), Lodja (Sankuru), and Boende (Tshuapa) were purposively selected for this survey (Fig 1). First, the city of Kinshasa, in Kinshasa province, is well documented as the DRC’s center of wild meat trade coming from surrounding provinces by roads and rivers. Although this city has not been affected by Ebola, it is facing the current Mpox outbreak [17,32]. Understanding risky behaviors and perception related to zoonoses associated with wild meat consumption in this city is critical since a zoonotic spillover can spread relatively quickly and reach global dimensions given its large population estimated at 14 million people and the connectivity of its airport to major cities in Africa, Europe, and the Middle East.

Second, Kindu is the capital city of Maniema Province, located in the eastern part of the country. It has an estimated population of approximately 500,000 inhabitants, with a population density of 4,481.2 inhabitants per square kilometer [31,33]. The city is well-connected to neighbouring countries in the East African region, facilitating regional interactions and trade. Third, Lodja, the second-largest city in Sankuru Province after Lusambo, is situated in the central part of the country and it has an estimated population of 700,000 inhabitants and a population density of 58 inhabitants per square kilometer [33]. Lastly, Boende, the capital city of Tshuapa Province, is located within the tropical Congo Basin forest region with an estimated population of 600,000 inhabitants and a population density of 1,538.1 inhabitants per square kilometer [31,33]. The inclusion of cities in Maniema, Sankuru, and Tshuapa Provinces in this study was informed by their endemic status for Mpox, a critical factor given the history of outbreaks in these regions [34–36].

### Population sampling, and recruitment

The study targeted adults (aged 18 years old and above) who lived in the study area for more than six months. Considering that approximately 95% of individuals reported wild meat consumption in a previous study in Sankuru province [37], we assumed similar likelihood of consumption in other provinces to estimate a minimum sample size of 196 for 80% power and a 5% significance level.

A web-based questionnaire in French (Supplementary material) was used for data collection.

Four data collection assistants were trained and deployed in each of the selected cities. They lived in these cities and has good knowledge of the local areas and languages. They were instructed to conduct interviews with individuals they encountered each day on the streets for 10 days. For each city, we purposively selected places with high pedestrian traffic, such as streets and traffic circles, to recruit study participants. After explaining the study objectives and obtained consent, participants were invited to respond to the survey questions and responses were recorded by the assistants.

### Data sources and measures

A three-section questionnaire was developed and hosted on Smart Survey [38]. The first section covered sociodemographic characteristics. The individual monthly income level, was self-assessed by the respondents based on one of the three proposed categories (low: ≤ USD$5,00; middle: > USD$5,00 ≤ USD$2,000; high income: > USD$2,000). The second section covered various topics related to wild meat consumption. We considered wild meat as animals killed from any species of vertebrate, regardless of size, that occurs in the region and is not subject to domestication or husbandry practices by humans. The respondents were asked to report if they consumed wild meat (yes or no), if yes when they last consumed the meat and indicate the animal species consumed, where they accessed the meat, and the reasons for consumption (with the ability for respondents to one or more options). Participants could choose from the following reasons for wild meat consumption, based on reasons previously reported in the literature [39]: accessibility (availability of the meat at the local market), affordable price, health benefits, tradition, meat taste, or prestige. In this study a friend is a person with whom you have a close, mutual bond formed through shared interests, experiences, or companionship. A relative is a person connected to you through blood, marriage, or legal ties (e.g., adoption). The following section was about the perception of the risk for acquiring a zoonotic disease following wild meat consumption. Participants were asked to report if they felt at risk of any zoonotic disease after consumption (yes or no), if yes, they were also asked to state the name of the disease and any known family member who suffered from one of the mentioned diseases. In this paper, the “not stated” in this study means the respondent did not provide a response to the question. In the last section, we adapted the Wildlife Conservation Society (WCS) proposed measures to control diseases from wildlife and asked participants to state their level of agreement using a Likert scale [40]. These measures included law enforcement, awareness campaigns, banning of wild meat consumption, and strengthening prevention and response (Supplementary material).

### Statistical analyses

The dataset was downloaded from the Smart Survey server as a csv file, and the R software Version 4.0.1 [41] was used for analysis. We summarized categorical variables by their relative frequencies and reported their percentage. We used multivariable analyses for exploring the association with wild meat consumption and risk perception. To refine the models, we applied a backward selection method, systematically removing non-significant variables to optimize model fit, as indicated by the Akaike Information Criterion (AIC) [42]. We also accounted for potential interactions between participants’ occupations and their income status, gender and marital status, recognizing that these factors could influence wild meat consumption. Additionally, we adjusted the model for city and other covariates to control for potential confounders. We report the adjusted odds ratios (ORs) and their corresponding 95% confidence intervals (CIs) and *p*-values for each variable included in the final model.

### Ethics statement

This study received ethical approval from the Kinshasa School of Public Health Ethical Review Board (ESP/CE/40/2022). Before accessing the survey, participants were presented with a digital information sheet detailing the study’s objectives and a confidentiality statement. Participants were required to read the study summary and provide electronic consent before proceeding.

## Results

### General characteristics of study sample

The survey included N = 2,163 respondents from four cities each from different provinces of the DRC. The age group between 26–35 years was the most represented with 37% of respondents overall, while in Kindu, the age group 45 years and above was the most represented (45%). More than half of the respondents were males (59%) and Lodja represented 32% of responses, followed by Kindu (27%). The majority of participants surveyed reported an income level from low to middle-income. The majority of respondents (86%) reported wild meat consumption (Table 1).

**Table 1:** Participants socio-demographic characteristics by city.

Variables	Overall N = 2,163	Kinshasa N = 352	Kindu N = 583	Lodja N = 696	Boende N = 532
**Age groups**					
18–25	304 (14%)	46 (13%)	40 (6.9%)	124 (18%)	94 (18%)
26–35	811 (37%)	175 (50%)	137 (23%)	253 (36%)	246 (46%)
36–45	524 (24%)	83 (24%)	142 (24%)	198 (28%)	101 (19%)
46 and above	524 (24%)	48 (14%)	264 (45%)	121 (17%)	91 (17%)
**Gender**					
Female	892 (41%)	153 (43%)	212 (36%)	295 (42%)	232 (44%)
Male	1,271 (59%)	199 (57%)	371 (64%)	401 (58%)	300 (56%)
**Level of education**					
No education	42 (1.9%)	1 (0.3%)	25 (4.3%)	9 (1.3%)	7 (1.3%)
Primary level	204 (9.4%)	12 (3.4%)	34 (5.8%)	88 (13%)	70 (13%)
Secondary level	1,319 (61%)	204 (58%)	216 (37%)	535 (77%)	364 (68%)
University level	598 (28%)	135 (38%)	308 (53%)	64 (9.2%)	91 (17%)
**Socio-economic level**					
High income	39 (1.8%)	3 (0.9%)	32 (5.5%)	0 (0%)	4 (0.8%)
Low income	1,127 (52%)	221 (63%)	180 (31%)	552 (79%)	174 (33%)
Middle income	997 (46%)	128 (36%)	371 (64%)	144 (21%)	354 (67%)
**Marital status**					
Divorced	57 (2.6%)	0 (0%)	41 (7.0%)	9 (1.3%)	7 (1.3%)
Married	1,411 (65%)	156 (44%)	359 (62%)	598 (86%)	298 (56%)
Single	630 (29%)	195 (55%)	143 (25%)	71 (10%)	221 (42%)
Widow-widower	65 (3.0%)	1 (0.3%)	40 (6.9%)	18 (2.6%)	6 (1.1%)
**Occupation status**					
Government employee	263 (12%)	54 (15%)	74 (13%)	64 (9.2%)	71 (13%)
Private sector employee	334 (15%)	88 (25%)	157 (27%)	65 (9.3%)	24 (4.5%)
Self employed	663 (31%)	171 (49%)	226 (39%)	113 (16%)	153 (29%)
Student	181 (8.4%)	15 (4.3%)	69 (12%)	53 (7.6%)	44 (8.3%)
nemployed	722 (33%)	24 (6.8%)	57 (9.8%)	401 (58%)	240 (45%)
**Wild meat consumption**					
Yes	1,866 (86%)	270 (77%)	488 (84%)	678 (97%)	430 (81%)

### Wild animal consumed, source of meat and risk perception of disease after consumption

Ungulates and primates were the most consumed meat with 37% and 33% respectively, the majority of respondents reported having consumed species from these taxonomic groups over a twelve-month period. Other animals cited as consumed by respondents included rats, pangolins, and tortoises (not shown in the table). Local markets were reported as the first source of wild meat consumed by respondents (53%) (Table 2).

**Table 2:** Animal consumed, source of wild meat by city.

	Overall N = 1,866	Kinshasa N = 270	Kindu N = 488	Lodja N = 678	Boende N = 430
**Monkeys**					
Yes	612 (33%)	65 (24%)	199 (41%)	180 (27%)	168 (39%)
**Snakes**					
Yes	80 (4.3%)	2 (0.7%)	31 (6.4%)	42 (6.2%)	5 (1.2%)
**Elephants**					
Yes	12 (0.6%)	7 (2.6%)	3 (0.6%)	2 (0.3%)	0 (0%)
**Antelopes**					
Yes	697 (37%)	44 (16%)	316 (65%)	130 (19%)	207 (48%)
**Bats**					
Yes	57 (3.1%)	0 (0%)	3 (0.6%)	45 (6.6%)	9 (2.1%)
**Source of the meat**					
Friend or relative	205 (11%)	9 (3.3%)	31 (6.4%)	152 (22%)	13 (3.0%)
Hunting by myself or a relative	181 (9.7%)	0 (0%)	16 (3.3%)	164 (24%)	1 (0.2%)
Local market	998 (53%)	74 (27%)	370 (76%)	192 (28%)	362 (84%)
Local restaurant	16 (0.9%)	11 (4.1%)	5 (1.0%)	0 (0%)	0 (0%)
Not stated	255 (14%)	160 (59%)	26 (5.3%)	56 (8.3%)	13 (3.0%)
Received from the village	211 (11%)	16 (5.9%)	40 (8.2%)	114 (17%)	41 (9.5%)
**Last time you consumed**					
This week	1,034 (55%)	45 (17%)	331 (68%)	345 (51%)	313 (73%)
Last week	405 (22%)	41 (15%)	95 (19%)	181 (27%)	88 (20%)
Last month	280 (15%)	67 (25%)	52 (11%)	134 (20%)	27 (6.3%)
Two months and above	147 (7.9%)	117 (43%)	10 (2.0%)	18 (2.7%)	2 (0.5%)

### Reasons for wild meat consumption

Out of the participants who reported wild meat consumption, the taste of the meat was the first reason (76%), followed by affordability (38%), and perceived health benefits (23%). In Lodja and Boende, wild meat was reported to have an affordable price by 62% and 38% of respondents respectively. In Boende, the meat was perceived to have health benefits by more than half of the participants (57%). On the other hand, only a low number of respondents mentioned accessibility, traditional reasons or prestige as reasons for consumption (Table 3).

**Table 3:** Reasons for wild meat consumption among consumers by city.

Reasons	Overall N = 1,866	Kinshasa N = 270	Kindu N = 488	Lodja N = 678	Boende N = 430
**Easy to access**					
Yes	138 (7.4%)	4 (1.5%)	34 (7.0%)	87 (13%)	13 (3.0%)
**Affordable price**					
Yes	706 (38%)	4 (1.5%)	118 (24%)	419 (62%)	165 (38%)
**Meat taste**					
Yes	1,416 (76%)	200 (74%)	426 (87%)	495 (73%)	295 (69%)
**For prestige**					
Yes	110 (5.9%)	67 (25%)	2 (0.4%)	35 (5.2%)	6 (1.4%)
**For traditional reasons**					
Yes	120 (6.4%)	14 (5.2%)	4 (0.8%)	89 (13%)	13 (3.0%)
**For health benefits**					
Yes	431 (23%)	65 (24%)	36 (7.4%)	86 (13%)	244 (57%)

### Perceived risk of disease after wild meat consumption

Overall, 36% of respondents perceived the risk of disease after wild meat consumption, with the highest level of perceived risk (92%) observed in Boende (Table 4). Mpox and Ebola were the diseases that most participants mentioned. Thirty-five percent of respondents who consumed wild meat reported knowing a person, either a family member or a relative, who suffered from a zoonotic disease, this percentage was as high as 93% in Boende. Overall, 40% of respondents reported that their wild meat consumption decreased in response to Ebola and/or COVID-19 impact, with the highest proportion of reduction mentioned in Kindu (66%) and Boende(48%) (Table 4).

**Table 4:** Perceived risk of disease after wild meat consumption by city.

	Overall N = 1,866	Kinshasa N = 270	Kindu N = 488	Lodja N = 678	Boende N = 430
**Risk of disease**					
No	1,195 (64%)	238 (88%)	429 (88%)	495 (73%)	33 (7.7%)
Yes	671 (36%)	32 (12%)	59 (12%)	183 (27%)	397 (92%)
**Which disease**					
COVID-19	22 (1.2%)	0 (0%)	0 (0%)	1 (0.1%)	21 (4.9%)
Ebola	182 (9.8%)	23 (8.5%)	19 (3.9%)	9 (1.3%)	131 (30%)
Mpox	453 (24%)	7 (2.6%)	35 (7.2%)	174 (26%)	237 (55%)
Not stated	1,194 (64%)	239 (89%)	429 (88%)	493 (73%)	33 (7.7%)
Others	11 (0.5%)	0 (0%)	3 (0.6%)	0 (0%)	8 (1.9%)
Rabies	4 (0.2%)	1 (0.4%)	2 (0.4%)	1 (0.1%)	0 (0%)
**Any family member or relative infected**					
No	1,211 (65%)	248 (92%)	474 (97%)	467 (69%)	22 (5.1%)
Yes	655 (35%)	22 (8.1%)	14 (2.9%)	211 (31%)	408 (95%)
**Which disease**					
COVID-19	22 (1.2%)	0 (0%)	0 (0%)	1 (0.1%)	21 (4.9%)
Ebola	182 (9.8%)	23 (8.5%)	19 (3.9%)	9 (1.3%)	131 (30%)
Mpox	453 (24%)	7 (2.6%)	35 (7.2%)	174 (26%)	237 (55%)
Not stated	1,194 (64%)	239 (89%)	429 (88%)	493 (73%)	33 (7.7%)
Others	11 (0.5%)	0 (0%)	3 (0.6%)	0 (0%)	8 (1.9%)
Rabies	4 (0.2%)	1 (0.4%)	2 (0.4%)	1 (0.1%)	0 (0%)
**Have these disease impacted your consumption?**					
I don’t eat wildmeat anymore	53 (2.8%)	9 (3.3%)	38 (7.8%)	0 (0%)	6 (1.4%)
I reduced my consumption	746 (40%)	34 (13%)	321 (66%)	185 (27%)	206 (48%)
My consumption has not changed	1,060 (57%)	226 (84%)	128 (26%)	489 (72%)	217 (50%)
My consumption increased	7 (0.4%)	1 (0.4%)	1 (0.2%)	4 (0.6%)	1 (0.2%)

### Factors associated with wild meat consumption

In the adjusted model (Table 5), residency in Lodja, in Sankuru province, remains strongly associated with wild meat consumption, with the highest odds ratio (OR: 11.4, CI: 6.35–21.4) compared to Kinshasa and Kindu (OR: 1.61, CI: 1.19–2.37). There was no significant association for levels of education, income level, and occupational status. Knowing a family member of a relative infected with a zoonotic disease was not significantly associated with wild meat consumption (Table 5).

**Table 5:** Adjusted association between wild meat consumption and sociodemographic characteristics.

Variables	Unadjusted				Adjusted		
	N	OR	95% CI	p-value	OR	95% CI	p-value
Age groups	2,163						
18-25		—	—		—	—	
26-35		0.69	0.46, 1.02	0.073	0.78	0.49, 1.21	0.3
36-45		0.75	0.48, 1.15	0.2	0.75	0.44, 1.26	0.3
46 and above		0.92	0.59, 1.43	0.7	0.98	0.56, 1.72	>0.9
Gender	2,163						
Female		—	—		—	—	
Male		1.13	0.88, 1.44	0.3	1.16	0.89, 1.51	0.3
City	2,163						
Kinshasa		—	—		—	—	
Kindu		1.56	1.12, 2.17	0.008	1.61	1.09, 2.37	0.016
Lodja		11.4	6.90, 20.0	<0.001	11.4	6.35, 21.4	<0.001
Boende		1.28	0.92, 1.78	0.14	1.50	0.82, 2.70	0.2
Level of education	2,163						
No education		—	—		—	—	
Primary level		0.21	0.01, 1.06	0.14	0.20	0.01, 1.05	0.13
Secondary level		0.18	0.01, 0.81	0.087	0.21	0.01, 1.00	0.13
University level		0.10	0.01, 0.48	0.026	0.18	0.01, 0.90	0.10
Socio-economic level	2,163						
Low income		—	—		—	—	
High income		0.33	0.16, 0.73	0.004	0.54	0.24, 1.30	0.2
Middle income		0.55	0.43, 0.71	<0.001	0.88	0.63, 1.24	0.5
Marital status	2,163						
Single		—	—		—	—	
Divorced		2.27	0.97, 6.64	0.087	1.32	0.52, 4.07	0.6
Married		1.56	1.21, 2.02	<0.001	0.93	0.66, 1.30	0.7
Widow-widower		2.15	0.98, 5.68	0.083	1.04	0.43, 2.95	>0.9
Occupation status	2,163						
Unemployed		—	—		—	—	
Government employee		0.56	0.37, 0.86	0.007	1.13	0.67, 1.93	0.6
Private sector employee		0.43	0.30, 0.63	<0.001	0.81	0.51, 1.29	0.4
Self employed		0.59	0.42, 0.82	0.002	1.05	0.71, 1.55	0.8
Student		0.49	0.31, 0.78	0.002	0.64	0.38, 1.11	0.11
Family member or relative	2,163						
No		—	—		—	—	
Yes		0.84	0.66, 1.09	0.2	0.81	0.49, 1.36	0.4

### Factors associated with disease risk perception

Disease risk perception was significantly higher in Boende (OR: 5.26,CI: 1.72–15.0). Participants reported significant lower risk perception (OR: 0.25, CI: 0.09–0.03) in Lodja compared to Kinshasa. Regarding marital status, we detected a lower risk perception especially for married women and widow/widower compared to single or unmarried respondents. Compared to unemployed participants, government employees showed a high likelihood of risk perception. Knowing a family member infected with a zoonotic disease event increased the likelihood of risk perception (OR: 5.55, CI: 2.29–13.4) as indicated in Table 6.

**Table 6:** Adjusted association between risk perception and demographic characteristics.

Variables	Unadjusted				Adjusted		
	N	OR	95% CI	p-value	OR	95% CI	p-value
Age groups	2,163						
18-25		—	—		—	—	
26-35		0.95	0.73, 1.25	0.7	1.02	0.58, 1.76	>0.9
36-45		0.69	0.51, 0.92	0.011	1.23	0.65, 2.36	0.5
46 and above		0.54	0.41, 0.73	<0.001	1.11	0.56, 2.16	0.8
Gender	2,163						
Female		—	—		—	—	
Male		0.95	0.80, 1.14	0.6	1.08	0.78, 1.52	0.6
Cities	2,163						
Kinshasa		—	—		—	—	
Kindu		1.10	0.75, 1.64	0.6	1.19	0.71, 2.04	0.5
Lodja		2.54	1.80, 3.66	<0.001	0.25	0.09, 0.60	0.003
Boende		75.7	49.4, 119	<0.001	5.26	1.72, 15.0	0.002
Level of education	2,163						
No education		—	—		—	—	
Primary level		2.04	0.96, 4.75	0.077	1.18	0.27, 5.10	0.8
Secondary level		2.37	1.17, 5.31	0.023	1.67	0.42, 6.65	0.5
University level		1.88	0.92, 4.26	0.10	2.21	0.52, 9.43	0.3
Socio-economic level	2,163						
Low income		—	—		—	—	
High income		0.86	0.39, 1.72	0.7	0.69	0.01, 41.8	0.9
Middle income		2.22	1.86, 2.66	<0.001	1.30	0.58, 2.96	0.5
Marital status	2,163						
Single		—	—		—	—	
Divorced		0.37	0.19, 0.68	0.002	0.73	0.23, 2.04	0.6
Married		0.69	0.57, 0.84	<0.001	0.40	0.25, 0.64	<0.001
Widow-widower		0.20	0.09, 0.40	<0.001	0.10	0.03, 0.37	<0.001
Occupation status	2,163						
Unemployed		—	—		—	—	
Government employee		1.52	1.15, 2.02	0.004	3.57	1.06, 12.0	0.041
Private sector employee		0.46	0.35, 0.62	<0.001	1.13	0.49, 2.57	0.8
Self employed		0.55	0.44, 0.69	<0.001	0.66	0.32, 1.34	0.3
Student		0.92	0.66, 1.28	0.6	2.00	0.90, 4.44	0.089
Wild meat consumption	2,163						
No		—	—		—	—	
Yes		0.70	0.55, 0.90	0.005	0.78	0.51, 1.21	0.3
Knowing a family member or relative infected	2,163						
No		—	—		—	—	
Yes		96.4	71.9, 131	<0.001	5.55	2.29, 13.4	<0.001

### Respondents’ opinion on proposed measures to control diseases from wildlife

**Fig 2:**
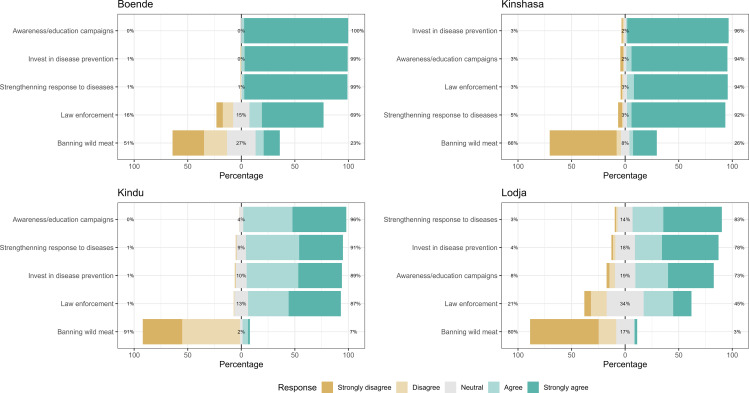
Participants’ opinions on proposed control measures for preventing zoonotic disease transmission by city. Stacked bar charts display the percentage of respondents in Boende, Kinshasa, Kindu, and Lodja who rated their level of agreement with five intervention strategies using a 5-point Likert scale (1 = strongly disagree to 5 = strongly agree). The proposed measures include awareness/education campaigns, investment in disease prevention, strengthening disease response, law enforcement, and banning wild meat. Sums may exceed one hundred because rounding.

The majority of participants were supportive of four out of the five proposed measures across cities with a similar trend across them. In Kinshasa, four measures received agreement from the majority of participants except banning wild meat consumption. In Kindu and Boende, the highest agreement was for awareness campaigns and lastly, in Lodja, the high agreement was toward increasing budget for outbreak response followed by strengthening disease prevention and awareness campaigns. Banning wild meat consumption received the least support across the four cities sampled (Fig 2).

## Discussion

This research is to our knowledge one of the first quantitative studies focused on the human dimensions of wild meat consumption and zoonoses in the DRC. We show that the highest proportion of wild meat consumers was found in Lodja, Sankuru province. Taste considerations were most commonly given as reasons for wild meat consumption, followed by affordability and perceived health benefits. Overall, less than half of respondents perceived risk of disease after wild meat consumption, with Boende reporting the highest level of risk perception compared to other cities. Participants who had personal experience with a zoonotic illness, either themselves or through a close relative, were more likely to perceive a risk related to wild meat consumption. The majority of participants agreed with four out of the five proposed measures to control zoonotic diseases including strengthening response to disease, improving disease prevention, education/ awareness campaigns, and law enforcement for wild consumption and trade. Banning wild meat consumption received the least support out of the proposed measures. In the adjusted model, residency in Lodja, in Sankuru province remained strongly associated with wild meat consumption.

Approximately two-thirds of the respondents in this study were under the age of 45, reflecting the demographic structure of the DRC population. More than half of the participants were male, and a significant proportion were married. In terms of socioeconomic status, most respondents were categorized as being in a lower economic bracket, with self-employment and salaried work being the most commonly reported occupations. These sociodemographic characteristics are consistent with national data on the DRC population, which is characterized by a high proportion of younger individuals and widespread informal employment. According to national statistics, the majority of the DRC’s population is under 45 years of age, and economic activities are predominantly concentrated on self-employment or small-scale enterprises [31].

This study noted a high proportion of wild meat consumption and significant difference across the study sites (p < 0,00), with the highest reported in Lodja, followed by Kindu and Boende. Meat taste, affordability and the perceived health benefits were among the most important reasons for consumption. High wild meat consumption proportion confirms previous findings [12,17,37,43]. Similar reasons were found in previous studies beyond the DRC, such as in Poland, United States and China, especially in relation to health benefits and taste preference [44–46]. Markets are supplied through informal supply chains since most hunted animals are legally protected, dynamic which is enabled by limited law enforcement [17,47]. There are several reasons explaining this highest consumption. First, these cities are part of the provinces which belong to the Congo basin forest with high biodiversity including animal species [48]. Second, these regions are facing economic disempowerment and therefore wild animal meat is considered as a source of income for several households which increases hunting and accessibility to the meat in the local markets [37,47,49]. Third, in the context of high food insecurity faced by these provinces including Kinshasa, wild meat is an important source of protein for these communities [43,47,50]. Wild meat consumption for cultural reasons did not emerge in this study despite being reported in previous studies [17,43,51]. This study involved urban residents in key cities where people may have weaker connection to their traditional cultural practices. Urbanization may reduce the cultural significance of wild meat consumption compared to rural settings, where cultural ties to such practices are often stronger [52].

Wild meat consumption is quite homogenous with regards to sociodemographic characteristics, only the city of residence emerged as a significant factor in the adjusted model. In a study conducted in China, higher income was significantly associated with wildlife meat consumption [46,53] in contrast to our findings. In the Chinese context, wild meat consumption is predominantly driven by cultural reasons and perceived health benefits [53], while in the DRC taste preference, affordability and food security are also drivers.

Our study found that there is a low perceived risk of disease overall. Boende, in Tshuapa province, reported the highest risk perception which could be potentially explained by Mpox related risk communication activities concurrent with the data collection period. People knowing family members or a relative infected by a zoonotic illness reported high risk perception as it allows better understanding of disease severity [54]. Previous studies reported low risk perception among actors working along the wild meat value chain in Kinshasa and Kisangani in the DRC [17,50,54]. They consider wild meat to be safe, and actors along the supply chains and consumers do not consider it to be a high-risk activity, even when they are in contact with meat products [37,50]. Risky behaviors identified in previous studies include butchering for sale, injuries while manipulating the meat for cooking or selling, transport, and using products for traditional medicine [37,55]. Marital status (especially married women and widow/widower) and occupation (unemployed and self-employed) were associated with lower risk perception. These two groups may experience limited access to information in the context of poverty which can impact their risk perception.

To control zoonotic diseases that may originate from hunting, trading, and consuming wild meat, participants in our study were supportive of education and awareness campaigns, strengthening prevention and response and law enforcement. The least support was noted for banning consumption as suggested by the Wildlife Conservation Society wildlife narrative of ending urban supply of wild meat [40]. Looking at the reasons provided above, it is of less cost to participants to implement measures which received high agreement. As mentioned above, banning wild meat in the context of poverty and food insecurity would have a negative impact on these communities [43,56]. The Wildlife Conservation Society calls for a One Health approach for preventing zoonotic disease originating from wildlife in central Africa. This requires alternative options to wild meat, such as farmed animal meat, messaging for improved understanding of risks and investing in early warning systems [8,40]. Addressing this issue is not only imperative from a public health and food security perspective, but also from a biodiversity conservation standpoint. The DRC is within a region with one of the highest diversities of primate species in the world, which are currently declining and at increased risk of extinction, with potential consequences to ecosystem function [57].

This study possesses several notable strengths. First, it addresses a significant gap in the limited body of research on wild meat consumption, disease risk perception, and strategies to control zoonotic diseases from wildlife in the DRC. Previous studies on these topics are predominantly qualitative or confined to very small areas, limiting their generalizability and ability to facilitate comparisons across cities. Second, in a vast and resource-constrained country such as the DRC, conducting data collection in four cities can be financially demanding. The methods employed in this study successfully achieved a large sample size while optimizing data collection costs. Third, the sample size of this study enabled comparisons across four cities, which are historically at heightened risk of zoonotic diseases from wildlife, including Mpox outbreaks. In this context, understanding risk perceptions and control strategies for zoonotic diseases has practical implications for improved planning and response efforts. Fourth, to mitigate potential selection bias, we employed a logistic regression model to adjust for covariates such as city of residence, gender, profession, and data collection methods. Additionally, presenting the results of zoonotic disease control measures disaggregated by province allowed us to account for inter-city variability.

Despite these strengths, the study has some limitations. The most critical limitation is the potential for selection bias stemming from the sampling method employed. First, using public places as data collection site cannot guarantee representativeness of the participants. Second, key sociodemographic characteristics, such as income levels, wild meat consumption status, and perceived disease risk, were self-reported by participants, introducing the potential for recall bias. Third, some of the risk of disease through wild meat consumption such as trapping, butchering and cooking were not directly included in the data collection questionnaire also people may have different risk perception according to different animal species, but our study did not assess this association. Finally, the study was conducted in cities, limiting the study’s ability to draw inference from, and comparisons with, rural populations where wild animal hunting is more prevalent. These limitations should be considered when interpreting our findings, particularly in the context of policymaking. Future research should aim to include rural populations, employ probability-based sampling methods, and use mixed methods to minimize biases and provide a more comprehensive understanding of zoonotic disease risk and prevention strategies in the DRC.

## Conclusion

In conclusion, this study sought to explore the determinants of wild meat consumption and strategies to control diseases from wildlife. Wild meat consumption proportion is very high among interviewed participants driven primarily by taste, affordability and perceived health benefits. Residency in Lodja, in Sankuru province, remained strongly associated with wild meat consumption. Controlling zoonotic diseases from wildlife requires strengthening response to disease system, improving disease prevention, implementing education/ awareness campaigns, and enforcing laws related to wild consumption and trade. Banning wild meat consumption received the least support out of the proposed measures. Furthermore, enhanced law enforcement, coupled with community-based education and alternative livelihood options, can help reduce dependence on wild meat and prevent zoonotic disease outbreaks.

## Supporting information

S1 File Inclusivity in global health questionnaire. Questionnaire completed by the research team to assess the inclusivity of the study design, implementation, and reporting in alignment with global health equity principles. (DOCX)

S2 File Data collection questionnaire. Structured questionnaire used for collecting data during the study, detailing variables related to wild meat consumption, disease risk perception and measures to prevent diseases from wildlife. (DOCX)
