# Supplementary material for: Awareness campaigns and strengthened prevention as alternatives to banning: Preventing zoonotic diseases from wildlife in the Democratic Republic of Congo
Source: PLoS One. 2025 Jul 1;20(7):e0327590. doi: 10.1371/journal.pone.0327590 (PMC12212513; doi:10.1371/journal.pone.0327590)
Supplement: S2 File — (DOCX) [file pone.0327590.s002.docx]

**Annex A Data collection questionnaire**

| **Number** | **Questions** | **Instruction** | **Jump to** |
| --- | --- | --- | --- |
| **Section 1: general information on the participant** | | | |
| Q101 | Age | Number (0-100) |  |
| Q102 | Gender   1. Male 2. Female 3. Others |  |  |
| Q103 | In which province do you live?   1. Kinshasa 2. Maniema 3. Sankuru 4. Tshopo | Select one |  |
| Q104 | Highest level of education   1. No education 2. Primary 3. Secondary 4. University level |  |  |
| Q105 | Marital status   1. Single 2. Married 3. Divorced 4. Widow-widower |  |  |
| Q106 | Employment condition:   1. Student 2. Unemployed 3. Self-employed 4. Work for a person, institution or company 5. Work for the government |  |  |
| Q107 | In which socio-economic level do you place yourself   1. Low income (<500 USD) 2. Middle income (501-2000 USD) 3. High income (>2001 USD) |  |  |
| **Section 2: Wild meet consumption** | | | |
| Q200 | Do you consume wild meat?   1. Yes 2. No | If no |  |
| Q201 | When was the last time you consumed wild meat during the last month?   1. This week 2. Last week 3. Last month 4. Two months and above |  |  |
| Q202 | Which animal meat have you consumed during the last month?   1. Monkey 2. Snake 3. Elephant 4. bat 5. Antelope 6. Other | If other, please specify |  |
| Q203 | How do you access the wild meat used for consumption?   1. Buy from the market 2. Sent to me from the village 3. Shared by a friend or colleague 4. Poaching from the bush 5. Please specify |  |  |
| **Section 3: Zoonotic disease risk perception** | | | |
| Q300 | Do you consider yourself being at risk of a zoonotic disease?   1. Yes 2. No |  |  |
| Q301 | Which zoonotic disease do you think you are at risk of?   1. Ebola virus disease 2. COVID-19 3. Monkeypox 4. Rabies 5. Other ………………………………………. | Please specify why/how ? |  |
| Q302 | Do you know any family member or relatives who suffered from a zoonotic disease?   1. Yes 2. No |  |  |
| Q303 | If yes, which one?   1. Ebola virus disease 2. COVID-19 3. Monkeypox 4. Rabies 5. Other ………………………………… |  |  |
| Q304 | For each the above-mentioned measures states your level of agreement using a 5 step Likert scale.  1 = Strongly disagree to 5 = Strongly agree   1. Law enforcement 2. Awareness campaigns 3. Strengthening prevention 4. Banning of wild meat consumption 5. Strengthening response | A value between 1-5 |  |

**Annex B Proposed intervention description**

| **N^o^** | **Interventions** | **Description** |
| --- | --- | --- |
| 1 | Law enforcement | Strengthen legal mechanism to reduce wildlife trade and consumption. |
| 2 | Awareness campaigns | Interventions to raise awareness on wildlife trade and consumption and risk of diseases from wildlife. |
| 3 | Banning of wild meat consumption | Interventions to stop wild meat consumption |
| 4 | Strengthening disease prevention | Interventions to strengthen wildlife disease prevention through early detection, and biosafety. |
| 5 | Increase budget for response | Interventions to strengthen the capacity to respond to diseases from wildlife. These include infrastructures, workforce capacity and financing. |
